# Dithiocarbamates: Properties, Methodological Approaches and Challenges to Their Control

**DOI:** 10.3390/toxics11100851

**Published:** 2023-10-11

**Authors:** Claudia Campanale, Mariangela Triozzi, Annamaria Ragonese, Daniela Losacco, Carmine Massarelli

**Affiliations:** CNR-IRSA, National Research Council of Italy, Water Research Institute, V.le F. De Blasio 5, 70132 Bari, Italy

**Keywords:** plant protection products, pesticides, carbamates, biomonitoring, agriculture, ethylene thiourea, propylene thiourea, green deal, toxicity, sustainability

## Abstract

Dithiocarbamates (DTCs) are a group of chemicals used primarily as fungicides, although they are exploited for various other applications. DTCs represent one of the oldest classes of broad-spectrum fungicides employed worldwide to control fungal diseases on many crops. Due to their ease of synthesis, low production costs (cheap and readily available starting materials) and a fungicidal activity with a multi-site mode of action, they are still among modern agriculture’s most extensively used pesticides. Although the environmental degradation in air, water, and soil is relatively rapid due to photolysis and/or hydrolysis, they are among the most frequently detected pesticides in the European Union (EU), also with a high frequency of maximum residue level (MRL) exceedances. The current review aims to comprehensively survey all aspects of DTCs, including the environmental fate, toxicity and analytical methods for determining parental compounds and degradation products in environmental and food samples. Furthermore, the accumulation of carbamate and dithiocarbamate pesticides in vegetables, fruits, bioindicator organisms and human biological samples, as well as their health effects on humans, are also considered in this study.

## 1. Introduction

Dithiocarbamates (DTCs), carbamate analogues in which sulphur atoms replace both oxygen atoms, are a group of organosulfur chemicals, of which 21 compounds are employed in agriculture as synthetic organic pesticides [1].

They are generated by the reaction of primary and secondary amines with carbon disulfide under alkaline conditions [1].

Their pesticide action is characterised by a broad spectrum of activity against various plant pathogens (above all fungi, but also bacteria, plants and insects) and low toxicity to mammals. The mechanism of action exploits their strong metal binding capacity (with Cu (II), Fe (II), Fe (III), Co (II), Mn (II), Ni (II) and Pb (II)) acting as enzyme inhibitors; indeed, they inhibit catalytic and regulatory thiol groups of cytoplasm constituents by organic electrophiles (isocyanates, carbonyl disulfide and isothiocyanates) generated by the metabolism of the parent molecules or by the coordinated metal ions (Zn(II) and Mn(II)) [2]. Therefore, their action on biological systems is improved in the form of heavy metal DTC salts [1,3].

DTCs can be classified into four subclasses according to their carbon skeleton:

❖ Methyl-dithiocarbamates (MDTCs), including metam sodium;
❖ Dimethyl-dithiocarbamates (DMDTCs), including ziram, thiram and ferbam;
❖ Ethylene-bis-dithiocarbamates (EBDTCs), including mancozeb, maneb, zineb and metiram;
❖ Propylene-bis-dithiocarbamates (PBDTCs), including propineb.

Their molecules contain a metal atom, which gives them their name. So, there are, for example, maneb, which contains Mn, ziram and zineb hold Zn and mancozeb, both Mn and Zn. Thiram is an example of a metal-free dithiocarbamate [4]. Metam sodium is the sodium salt of MDTCs, propineb is the zinc salt of PBDTCs as well as dazomet and milneb (thiadiazin) are in the form of heterocyclic six-membered ring(s) of methylene DTC and EBDTCs, respectively, and polycarbamate is a polymeric zinc salt of DMDTC and EBDTC [5,6].

Most of the DTCs are polymeric in nature; they have low solubility in water and most organic solvents (except for sodium salts, which are very soluble in water but rather insoluble in non-polar solvents), which makes their extraction from vegetable samples challenging using solvents [5].

In this context, the current review covers a critical compilation of all aspects concerning DTCs and their degradation products. It includes a description of analytical methodologies used for monitoring different samples, including conventional techniques routinely used and more advanced ones developed during the last years.

## 2. Materials and Methods

To carry out the current literature review, a research of references was obtained from the Web of Science (WoS) and Scopus databases. The considered works include only the research articles and reviews in English and published in peer-reviewed journals with impact factors greater than three. Moreover, the reports on pesticide residues in food of the European Food Safety Agency (EFSA) and the World Health Organization (WHO) reports for the classification of pesticides by hazard have also been consulted. The titles, abstracts and keywords were manually examined to exclude non-relevant articles.

## 3. Uses and Applications

Dithiocarbamates are versatile compounds used in various applications, mainly in agriculture as insecticides, herbicides and fungicides to protect fruits and vegetables and medicine.

**Figure 1:**
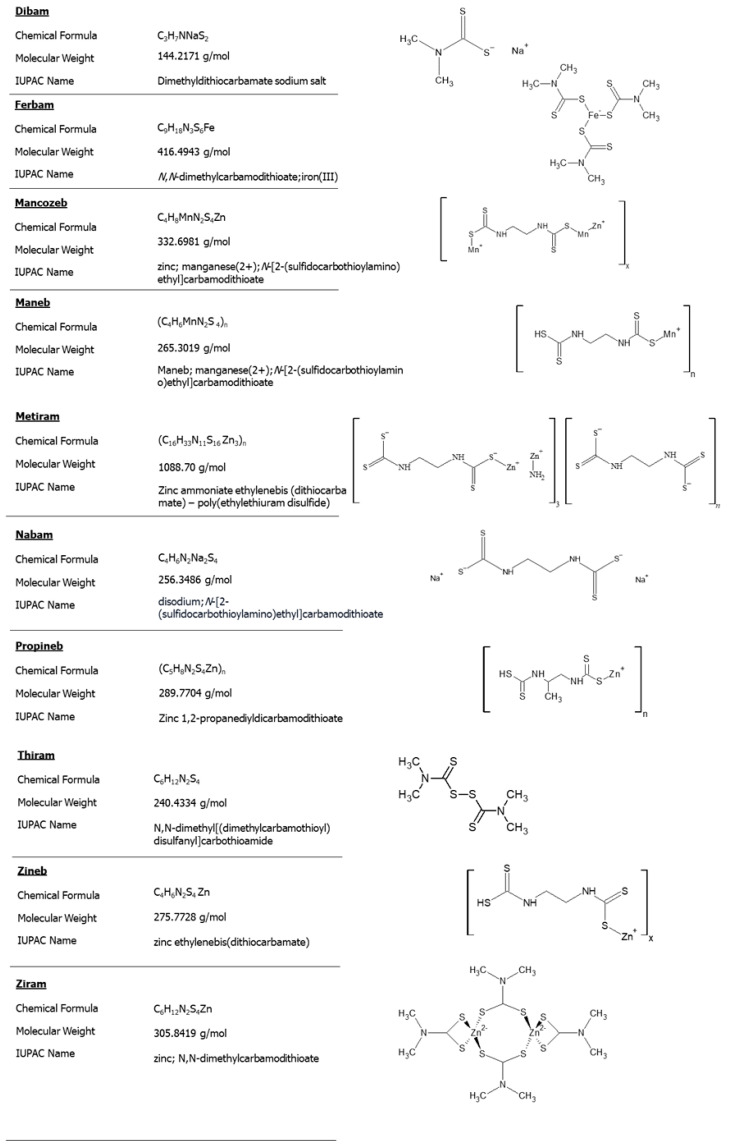
Chemical and structural formulas of main dithiocarbamates used in agriculture. X and Y are integers greater than 1. Data source: [7].

Some of the most-used dithiocarbamates in agriculture are dibam, ferbam, mancozeb, maneb, metam sodium, nabam, thiram, zineb and ziram (Figure 1) [3]. As pesticides, DTCs have been used in agriculture for over fifty years, with the first products, thiram and ziram, introduced to the market in the 1930s [6].

However, in 1945–1955, the real beginning of the pesticide industry with the so-called “second generation of pesticides” started with developing most organophosphorus insecticides and many carbamates [8].

**Figure 2:**
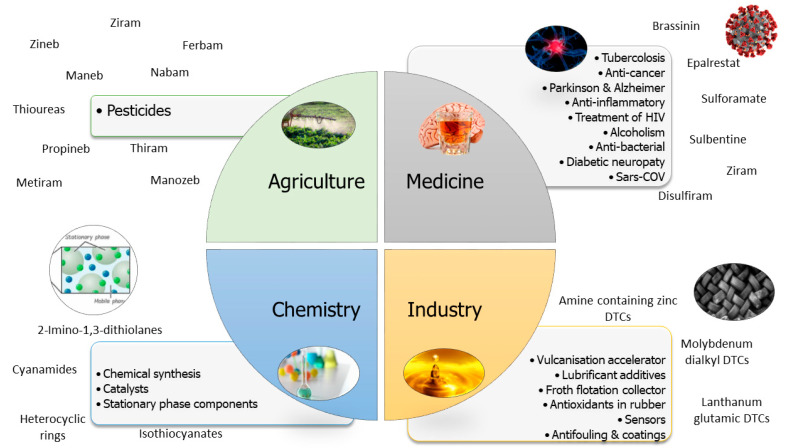
Main applications and primary dithiocarbamates used in the agricultural, medical, industrial and chemical fields.

Dithiocarbamates are widely used pesticides worldwide and are most frequently detected in monitoring programs [1,9]. Due to the broad range of applications (Figure 2), they are widely produced, with a yearly consumption of 25,000 and 35,000 metric tons worldwide [1]. 

Some act by killing the larvae of various parasites that cause plant diseases; others are used against nematodes, as they inhibit the hatching of the eggs and thus decrease their growth. Dithiocarbamate-based herbicides are used to prevent the growth of some broadleaf weeds; diallate, for example, controls monocotyledonous weeds and attacks their fatty acids.

In addition, they have been used as stationary phase components in ligand exchange chromatography due to their strong chelating ability; DTCs coated on silica-based stationary phase were used by [10] for heavy metal separation. Furthermore, they have been exploited as catalysts in organic transformations; core/shell nanostructures functionalised with magnetic DTCs have been used to synthesise propargyl amines [11,12,13,14].

The use of DTCs in the medical field has been investigated for more than 40 years due to their ability to bind metals and their high reactivity to other moieties (such as thiol groups).

The medical applications include:

(i) Anticancer. Compounds containing dithiocarbamates have been evaluated as anticancer drugs as they can inhibit catalase (an enzyme responsible for cancer growth) and induce apoptosis in the mitochondria [15]. For example, a pyrrolidine dithiocarbamate (PyDT)-zinc(II) complex and a PyDT-copper(II) complex were compared to treat breast and prostate cancer. Of the two, the copper complex was more potent in inhibiting the proteasome and inducing apoptosis [16]. Gold(III) compounds are also used as anticancer agents, and the characteristics of some gold(III) dithiocarbamate derivatives with cisplatin were compared. They are more cytotoxic, highly reactive towards some biological macromolecules and inhibit DNA and RNA synthesis much faster than cisplatin [17,18,19].
(ii) Alcoholism. The best-known DTC derivative is the diethyldithiocarbamate disulfiram [15]. Disulfiram (tetraethylthiuram disulfide) is a drug that has been used for 60 years to treat alcoholism as an aldehyde dehydrogenase inhibitor, which leads to the accumulation of acetaldehyde in the blood [20,21]. The main adverse side effects include flushing, nausea and tachycardia. Disulfiram also acts on the central nervous system by inhibiting dopamine-β-hydroxylase, which causes an increase in dopamine concentration in the brain. This can cause schizophrenia and, rarely, psychosis in otherwise healthy individuals [22]. However, the results obtained from the use of disulfiram are conflicting. In a controlled context, the results obtained are positive, even if it is often prescribed for short periods. The reasons are not entirely known, but the researchers think the side effects are usually minor and severe unpleasant reactions are uncommon, although monitoring should be undertaken [20,23,24].
(iii) Treatment of tuberculosis. Some N-mono- and N,N-di-substituted dithiocarbamates have been used to treat tuberculosis, acting through inhibition of the enzyme carbonic anhydrase. Both enzymes, mtCA 1 (Rv1284) and mtCA 3 (Rv3273), were inhibited using dithiocarbamates of formula R_1_R_2_N-CSSM where R_1_ is H, alkyl and substituted alkyl; R_2_ is alkyl, aryl and heterocycle and M is Na, K or triethylammonium. These DTCs were more effective than commonly used drugs [25,26].
(iv) Alzheimer’s treatment. Several coumarin–dithiocarbamate hybrids have been synthesised and evaluated to treat Alzheimer′s disease. Several compounds were tested, and it was found that the terminal amino group associated with the dithiocarbamate moiety inhibit acetylcholinesterase (AChE), and the cyclic amine substituents have more potent activity than the alkyl amines [15,27]. At the same time, the piperidinyl group proved to be more beneficial than the pyrrolidinyl group. However, the best compound is obtained with a four-carbon linker between coumarin and the dithiocarbamates′ fraction. The compound obtained has the maximum ability to inhibit the enzyme. It could interact simultaneously with the catalytic active site (CAS) and the peripheral anionic site (PAS) of AChE, reversing cognitive dysfunction [28].
(v) SARS-CoV-2 treatment. Dithiocarbamates have been used to treat MERS (Middle East respiratory syndrome) and SARS (severe acute respiratory syndrome) coronaviruses [29,30]. Specifically, disulfiram, a drug used to treat alcoholism, has been shown to inactivate viral coronavirus proteins (thioprotease and RNA replicase) [15]. Both disulfiram and some of its derivatives (tyram and dipentamethylenethiuram disulfide (DPTD)) are capable of inhibiting PLpro, a papain-like protease, through allosteric inhibition [31].

Finally, DTCs have been exploited in the industrial field as slimicides in water-cooling systems, sugar, pulp, paper manufacturing, vulcanisation accelerators, antioxidants in rubber, sensors or additives for lubricants [21,32,33].

### Latest Trends in the World Usage of Dithiocarbamates

The evaluation of the fate and potential effects of the plant protection products’ (PPPs) residues in ecosystems requires a consideration of the practical usage of the products applied in the present and in the past.

The estimation of the annual consumption of DTC reports a utilisation between 25,000 and 35,000 metric tons [34]. Mancozeb, propineb and thiram are among the top-selling fungicides, and mancozeb sales are expected to reach 18 billion dollars by 2025 [35].

**Figure 3:**
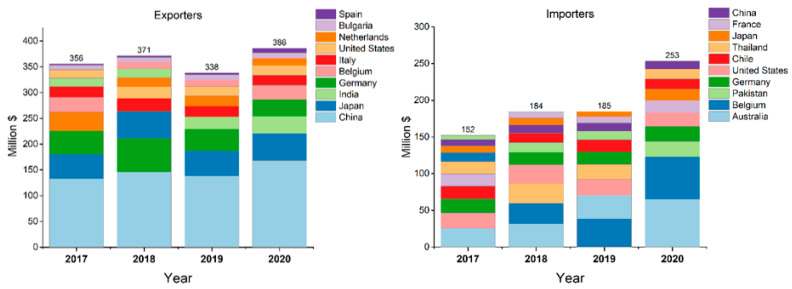
Importers and exporters of thiocarbamates and dithiocarbamates.

A general market trend showed among the top exporters of thiocarbamates and dithiocarbamates from 2017 to 2020 in China (with 168 million $ in 2020), followed by Japan (52.5 million $ in 2020) and India (33.4 million $) and Germany (32.8 million $). Conversely, among the importers in 2020, we found in first place Australia with 65 million $, followed by Belgium (58 million $), Pakistan (21 million $) and Germany (20 million $) [36] (Figure 3). Country importers′ variations may be explained by different EU regulations to which PPP are subjected to [37].

## 4. Metabolism and Environmental Fate

**Figure 4:**
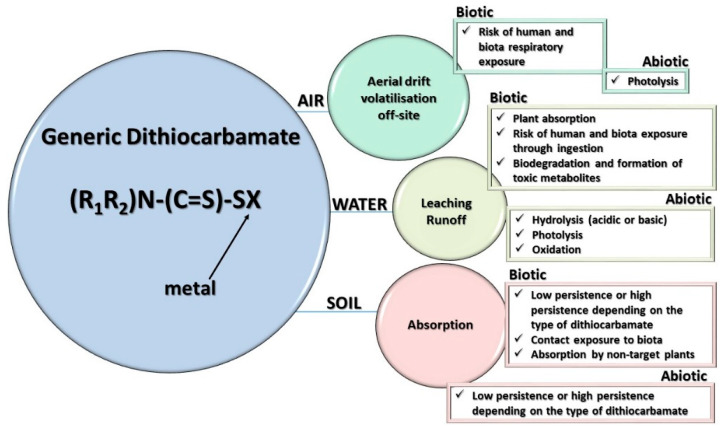
Dithiocarbamates environmental fate and degradation mechanisms occurring in the environment.

In order to assess the potential impact of PPPs, it is essential to evaluate their environmental fate (Figure 4) and distribution in soil, water and air matrices, considering, at the same time, the principal transformation products and their toxicity [8]. 

After pesticide application, a considerable amount remains associated with the soil particles over different periods depending on the compound physicochemical properties (especially Koc) and the soil and climatic conditions (especially the organic carbon content of the soil) [8].

Several processes are known to occur in soil, determining the environment′s pesticide fate; these include leaching, adsorption, absorption, volatilisation, photodecomposition, chemical reaction and metabolism by plants and soil microorganisms [38,39].

Alkyl dithiocarbamates (MDTCs and DMDTCs) are stable in an alkaline medium; the DMDCs are assumed to undergo acid-catalysed hydrolysis as the primary degradation route. The half-life at 25 °C can range from 2 h to 10 d depending on several factors, including pH, type of cation, climatic conditions and environmental matrix [40,41].

Conversely, EBCDs are susceptible to both acid-catalysed hydrolysis and oxidation based on pH values and oxygen concentration [40]; they are generally unstable in the presence of moisture, oxygen or biological systems, decomposing rapidly in water [21].

Some authors reported low to moderate DTC mobility in soil [42], and researchers discovered that soil microorganisms are involved in the carbamate and dithiocarbamate degradation acting as carbon and nitrogen sources [38,39,43]. Several microbial species belonging to the genera *Pseudomonas*, *Stenotrophomonas*, *Micrococcus*, *Enterobacter*, *Nocardioides*, *Pseudaminobacter*, *Serratia*, *Mucor*, *Trametes*, *Trichoderma*, *Pichia* and *Aspergillus* have been identified as involved in the DTC degradation processes that is enhanced by environmental conditions such as high temperature and moisture content [38].

In the atmospheric compartment, the DTCs mainly attach to air particulates, as their vapour pressures are very low. Similarly, in the aquatic compartment, carbamates and, therefore, DTCs are known to adsorb onto particles and sediments [42]; furthermore, they are expected to absorb light, thus undergoing rapid photolytic degradation [44]. However, DMDCs’ ability to form complexes with transition metals reduces their aqueous solubility and stabilises them, increasing their half-lives [40].

For example, some authors [45] identified the degradation of thiram in water and soil, reporting a fast transformation by hydrolysis, photolysis and oxidation, which produce many breakdown products.

**Figure 5:**
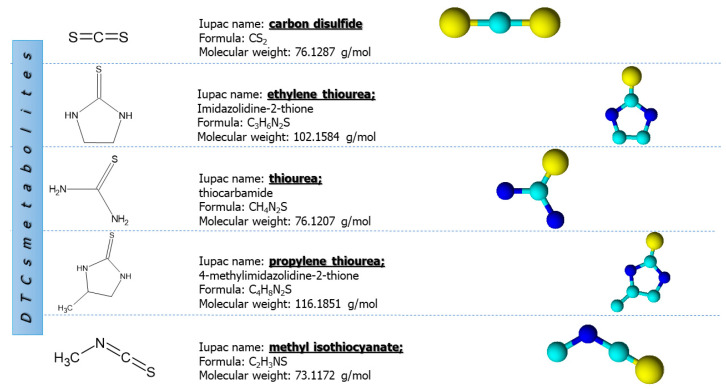
Structural and chemical formulas of main dithiocarbamate metabolites. Data source: [47].

Indeed, by splitting off carbon disulfide and hydrogen sulphide, as well as by oxidative degradation, dithiocarbamates are quickly metabolised into several metabolites such as ethylene thiourea (ETU), propylene thiourea (PTU) thiourea (TU), methyl isothiocyanate (MITC) and carbon disulfide (CS_2_) (Figure 5) [46].

Ethylene thiourea (ETU) is the primary degradation product of EBCD manufacturing, and it is formed in the presence of oxygen and moisture in EBCD formulations. ETU is water soluble and mobile and is absorbed by plant roots, translocated and metabolised, forming ethylene urea (EU) as well as other derivatives of 2-imidazole and various unidentified metabolites. In addition, in the presence of photosensitisers, ETU is easily photooxidised into EU [21]. Due to the EBDCs metabolic decomposition, ETU is also a by-product in mammals together with other metabolic products such as carbon disulfide, EDA, a few ethylene bisthiuram disulfides, hydrogen sulphide and ethylene bisthiocyanate. ETU is successfully split into moieties incorporated into molecules such as oxalic acid, glycine, urea and lactose [21]. Commercially, ETU is used in dyes, pharmaceutical products and synthetic resins [42]. Similarly, PTU and MITC are the by-products of propylene-bis-dithiocarbamates and metam and dazomet, respectively.

## 5. Toxicity of DTCs and Their Metabolites

Some dithiocarbamates have been classified by the World Health Organization (WHO) as hazardous compounds [48], and it is known that their abuse can pose severe risks to human health.

In general, the toxicity mechanisms of pesticides include their ability to generate a toxic molecule or a reactive intermediate and the ability to mimic a common biologically active molecule that interferes with normal cell homeostasis [49].

For DTCs, their toxicity is primarily related to the acetylcholinesterase (AChE) enzyme inhibition; indeed, some DTCs present a similar structure to this enzyme of the nervous system, which normally hydrolyses acetylcholine to acetic acid and choline, leading to the cessation of neurotransmitter signalling [39]. Carbamates bind to acetylcholine, reversibly inhibiting the acetylcholinesterase enzyme, which leads to the accumulation of acetylcholine levels, increasing neurotransmitter signalling [2,50].

Many short- and long-term toxicity studies (acute and chronic) have also been conducted on several dithiocarbamates, assessing the concentration causing the death of 50% of the test population (limit-dose 50 (LD_50_)) represented by mg/kg body weight. In rats, high doses of DTCs (>100 mg/kg body weight in mice and >200 mg/kg body weight in rats) evidenced problems in the reproductive and endocrine system and thyroid gland. Pituitary thyroid stimulation is caused by a low blood level of thyroxine, whose synthesis is inhibited by DTCs. Concentrations above 50 mg/kg can cause neurotoxic effects in rats and rabbits, paralysis and muscle atrophy in birds.

**Figure 6:**
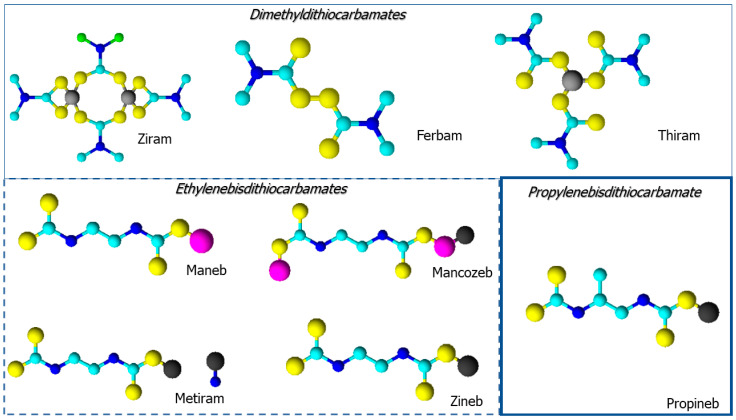
Dithiocarbamated 3D structural formulas.

Some in vivo tests conducted in rats have also shown the capacity of some DMTCs (ziram, ferbam, thiram) and EBDCs (Figure 6) to generate carbon disulfide (CS_2_) after oral dosing. Carbon disulfide is an important agent responsible for DTCs neuropathy. Indeed, degeneration and/or demyelination of sciatic or spinal nerve tissue was observed in rats exposed to mancozeb, maneb, metiram, ziram and thiram.

The toxicity of dithiocarbamates, together with parental compounds, is also attributed to their metabolic products [5]; ethylene and propylene thiourea (ETU and PTU) metabolites, together with carbon disulfide, maneb, mancozeb and metiram, are considered of concern due to their known thyroid toxicity in laboratory animals [1]. According to the International Agency for Research on Cancer (IARC), ETU was classified in 2001 as “Unclassifiable as to carcinogenicity in humans (Group 3)” due to the absence of carcinogenicity evidence in humans and sufficient evidence in the experimental animals [51]. However, the last report, dated 2021, of the U.S. National Toxicology Program (NTP) [52] classified ETU as “Reasonably Anticipated To Be Human Carcinogens”; this classification means a “limited evidence of carcinogenicity from studies in humans” or “a sufficient evidence of carcinogenicity from studies in experimental animals”, otherwise, “a less than sufficient evidence of carcinogenicity in humans or laboratory animals”.

ETU is assimilated orally and dermally. Its half-life in humans is approximately 17–23 h [53,54], and up to 90% is excreted in the urine [55].

Moreover, ETU is shown to be teratogenic, carcinogenic, mutagenic and immunotoxic in animals [42]. As well as ETU and PTU, methyl isothiocyanate is also more toxic than the parental molecule, and the European Food Safety Authority stated that DMTU should be regarded as toxicologically relevant [56].

Several studies have also shown that DTCs can cause neurotoxicity by mechanisms not involving ETU but rather due to the chelation of physiologically important polyvalent cations. The chelation can affect copper, zinc, cadmium and lead, leading to the formation of lipophilic species that may be involved in the distribution of heavy metals into the brain; this change of the distribution has also been found associated with peripherical neuropathies due to CS_2_ exposure [49].

In humans, prolonged or chronic exposition to DTCs, occurring by dermal contact, inhalation and ingestion, can lead to functional changes in the nervous and hepatobiliary systems, hormonal and reproductive disorders, immunotoxicity and cytotoxicity [2]. Regular contact can induce dermatitis, sensitisation, inflammation in the eyes and respiratory tract and skin allergy in occupational workers [2,21]. Some studies have shown that populations worldwide are exposed to DTCs; the analyses were performed by measuring the concentration of ETU in the urine. Median urine ETU levels ranged from 0.15 to 4.7 μg/g creatinine in adults (1994–2017), 0.24–0.83 μg/g creatinine in children (2011) and 2.6–5.24 ng/mL in pregnant women [57].

## 6. Analytical Methods for Dithiocarbamate Detection

DTCs represent one of the most frequently detected classes of plant protection products in the European Union. Various methods have been developed to analyse their residues and metabolites in food and environmental matrices [6].

DTCs constitute a complex pesticide group to be analytically determined due to their poor stability in vegetable matrices and low solubility in water and common organic extraction solvents. Several parameters, including temperature and pH, influence DTC analysis. Therefore, food and environmental monitoring requires the development of specific methods not compatible with multi-residue ones commonly used for the routine quantification of many plant protection product residues [41]. 

The analysis of food matrices requires the homogenisation of plant samples and the DTC extraction with organic solvents. Indeed, once DTCs come into contact with vegetable acid juices, they rapidly degrade and decompose into carbon disulfide (CS_2_) and the respective amine [58]. Still, many methods are, in fact, based on the detection of CS_2_ evolved after the acidic digestion of any dithiocarbamates present in the sample and its following detection by different techniques such as UV-Vis spectrophotometry, gas chromatography (GC) coupled to mass spectrometry or headspace GC [59,60,61,62].

Other authors have considered special techniques such as capillary electrophoresis and reverse-phase high-performance liquid chromatography (HPLC) coupled with optical, electrochemical or mass-spectrometry detectors [46] to focus on the detection of some single DTCs such as thiram [63], propineb and ziram [61,64].

Other methods include the use of biosensors based on enzymatic inhibition, stir bar sorptive extraction (SBSE), dispersant liquid–liquid microextraction (DLLME), solid-phase extraction (SPE) and solid-phase microextraction spectroscopy (SPME) [65], and Raman spectroscopy [46,66].

### 6.1. Hot Acid Digestion-Based Methods

Many methods developed to analyse DTCs in different matrices are based on the official EPA method 630 [67], a colourimetric method applicable to determining DTC pesticides in municipal and industrial wastewater. The method is based on reducing DTC compounds to carbon disulfide released during hot acid digestion; the total dithiocarbamate concentration is measured from the amount of CS_2_ produced and measured by spectrophotometric techniques.

The total DTCs concentration is expressed as the sum of CS_2_, but fails to distinguish among the individual analytes. An aliquot of the sample of approximately 1 L is digested in a hydrolysis flask with a tin chloride solution dissolved in concentrated hydrochloric acid; bringing the liquid in the flask to a gentle boil, the reaction leads to the formation of carbon disulfide by hydrolysis of the dithiocarbamate moiety. The developed CS_2_ is conveyed by a slight vacuum/stream of nitrogen into the hydrolysis apparatus. The CS_2_ evolved is then subjected to purification; it is then absorbed in an ethanol solution and reaction with copper acetate in the presence of diethanolamine leads to the formation of a yellow complex [58]. The absorbance of the coloured complex can be measured at 435 nm using a UV–visible spectrophotometer [6] or, alternatively, CS_2_ evolved from the acid treatment of the sample can be analysed by gas chromatography technique [68].

Method interferences may occur by contaminants in reagents, glassware and laboratory equipment; therefore, it is also essential to correctly evaluate the washing reagents and their power to eliminate any interference. The pairs of reagents proposed by the different authors, e.g., lead and sulphuric acid acetate, sodium hydroxide and sulphuric acid or sodium hydroxide and lead acetate, are environmentally and economically viable. Sulphuric acid has also been effective in reducing background interference [58].

Additional matrix interferences due to the formation of collateral compounds may also occur in the DTC analysis. For example, the acid hydrolysis of thiram (tetramethylthiram disulfide, TMTD) leads to the formation of reaction products carbonyl sulphide (COS) and hydrogen sulphide (H_2_S) at the expense of CS_2_ [69]. Amines may be used as absorbent agents to separate CS_2_ and other reaction products [13]. It is important that the digestion/distillation flask′s temperature reaches boiling point faster. In addition, a significant problem concerns the quantification of phytogenic CS_2_, which leads to false positives. In fact, plants of food interest, such as *Brassica*, produce glucosinolates [70]. Such natural substances, during the acidic digestion of DTCs, can produce CS_2_ and thus can lead to an overestimation of the content of DTCs in these agricultural products [5]. This is a pervasive problem in raw materials used to prepare baby food [5].

Some authors have tried to evaluate the specific ranges of CS_2_ naturally produced by *Brassica* under conditions of acid digestion. The evaluations were carried out during post-harvest treatments or in the processing of horticultural products belonging to this crop [71]. The researchers noted that natural plant components such as brassine or isothiocyanates favour the formation of CS_2_ under certain conditions. Other authors evaluated the effect of the sulphurisation and sulphur residues on the traditional DTCs analysis method. Various agricultural foods are subjected to the treatment of sulphurisation that improves the use of the product, but, at the same time, it releases sulphur residues [35]. The evaluation was conducted on sulphur dioxide and dithiocarbamates present in apricots before and after the sulphurisation process. The study shows that measuring total CS_2_ as the sum of DTCs after sulphurisation can lead to false positives because sulphur compounds (or also pesticides) are present in products and can be considered DTCs by mistake [72]. Consequently, the formation of CS_2_ is not an unequivocal indication of the presence of the DTCs [71].

Therefore, CS_2_ may not always be well correlated with DCTs and identifying the specific residues of this pesticide group would be necessary. Indeed, the European Food Safety Agency (EFSA) has expressed the need for analytical protocols specific to each active ingredient of the DTCs.

In a study of [73], the researchers proposed a new digestion/distillation protocol to overcome the interferences issues, with washing and absorption units positioned vertically on the reflux condenser. The apparatus was easier to assemble and less vulnerable to gas leaks from the joint points than the traditional one cited in the EPA method [73]. Moreover, the new vertical arrangement allowed a percentage of recovery of ziram, mancozeb and thiram extracted from plant matrices ranging from 82% to 120%. However, in other similar matrices, recoveries appeared to be the same as those obtained using the traditional digestion/distillation system [73].

Subsequently, attention was placed on the efficiency of washing in the vertical system, which improved the sensitivity of the method, particularly for the matrices subjected to background interference [74]. Some authors used a modified vertical system in which the two washing chambers (arranged vertically) with a sintered glass bottom contain approximately 10 g of boiling chips wet with 50% concentrated solutions of NaOH and H_2_SO_4_. A methanol KOH solution absorbs the gas. The system described allowed them to reach the limit of quantification (LOQ) in fruits and vegetables up to 0.01 mg/kg^−1^ of CS_2_ [74].

### 6.2. Gas Chromatography-Based Methods

Although spectrophotometric determination is still widely used in DTC analysis, the chromatographic approach may become the accepted method because it allows better recoveries and high sensitivity [75]. Gas chromatography coupled to mass spectrometry (GC-MS) is considered one of the techniques indicated in the most widely used and standardised methods (EN 12396) for analysing dithiocarbamate fungicides.

The CS_2_ derived from DTCs digestion was measured by gas chromatographic [68] coupled with more selective detectors such as electron capture, pulsed flame photometric and ion trap mass spectrometry detectors (GC-ECD, GC-PFPD and GC-ITD-MS). In a study of [75], the authors determined DTCs as the sum of CS_2_ in soya by GC-PFPD and GC-ITD-MS. The method showed good recoveries (between 68 and 91%) and LOQ equal to 0.05 mg kg^−1^ of CS_2_ [75,76].

Another study conducted by [77] reported a study in which the authors determined separately millinebs, ethylene bisdithiocarbamate (EBDTC), propylene bisdithiocarbamate (PBDTC) and dimethyldithiocarbamate (DMDTC). The technique used was GC-MS after extracting fungicides from plant and zootechnical matrices in an alkaline ethylenediaminetetracetic acid (EDTA)/cysteine solution. DTCs were derived in their methyl esters with methyl iodide (LOQ 72 mg/kg) [77].

The analysis of individual DTCs in tap water is rather complex. Several methods have been developed (capillary-UV electrophoresis, HPLC-UV, HPLC ion pair, etc.) but need better selectivity and sensitivity. Some authors [76] analysed the polycarbamate, consisting of EBDC combined with zinc and DMDC, with a GC-MS method. The method involves the programmed injection at the temperature of the input column. The compound was analysed as methyl derivatives of dimethyldithio-carbamate (DMDC) and dimethyl ethylene bisdithiocarbamate (EBDC). The technique makes it possible to measure the polycarbamate in tap water better. In addition, the simultaneous analysis of DMDC-methyl and EBDC-dimethyl allows recognition from other DTCs, such as thiram and ziram, with DMDC side chains. The analysis showed a recovery of 79% with an RSD of approximately 6% [76].

### 6.3. Liquid Chromatography-Based Methods

Dithiocarbamates include neutral compounds such as thiram (dimethylf-thiocarbamate) and disulfiram, which are more easily analysed than the other ones due to their tendency to create complexes with different metals and polymers. Several methods detect DTCs ions after their complexation with specific agents such as ethylenediaminotetraacetic acid (EDTA).

Some authors analysed ziram, thiram and zineb by extraction and subsequent high-performance liquid chromatography coupled to a UV detector (HPLC-UV) on a reverse-phase column (Nucleosil RP-18). In the method, ziram and zineb were subjected to a double extraction from the vegetable matrix; the first extraction was performed through an EDTA/cysteine solution and the second one with an organic solvent (tetrabutylammonium hydrogen sulphate) for a final derivatisation with methyl iodide. Otherwise, thiram was extracted with chloroform and analysed by direct injection with HPLC-UV. Good recoveries were obtained for different crops and water samples (59–85%) with a limit of detection (LOD) equal to 0.01 mg/kg for ziram, 0.02 mg/kg for zineb and 0.01 mg/kg for thiram [58,78].

Other authors tried to extract thiram from soil and foods (apples and lettuce), with a dichloromethane solution. The analyte was loaded onto a reverse-phase column and then complexed with copper (II). Differently, zineb and maneb were extracted from the soil with an alkaline solution of EDTA, selectively pre-concentrated as ion pairs on a pre-column C18 and loaded online with cetyltrimethylammonium bromide. Thiram showed a LOD ranging from 0.005 to 0.01 mg/Kg for soil and apples, while lettuce showed a LOD equal to 0.05–0.1 mg/Kg [79].

Two different procedures have been described by [80], to quantify ziram in spinach grown in greenhouses. Both require determination with HPLC and ultraviolet detection. In both cases, the samples were freeze dried; in the first protocol, the sample was extracted with supercritical carbon dioxide, adding methanol as an organic modifier. In the second method, the extraction was performed using an EDTA–methanol solution (1:1). On the one hand, the extraction with supercritical carbon dioxide resulted acceptable for low ziram concentrations and a small amount of samples. On the other hand, the EDTA-methane-based extraction was more suitable for samples with a higher content of ziram [80].

Van Lishaut and Schwack [81] proposed the first work to detect four classes of DTCs (ethylene dithiocarbamates, N-methylthiocaramates, N,N-dimethyldithiocarbamates, propylene dithiocarbamates) in plant samples using a reverse-phase ion-pair chromatography method coupled to a UV and electrochemical detection. Several plant matrices showed recoveries ranging from 72 to 111% and LOD of 4–8 µg/L.

Perz and Schwack [82] added cysteine to the alkaline extracts of the samples to increase the stability of the DTCs. They also optimised the method of Van Lishaut and Schwack [81], using a new column, Xterra RP18. The method showed good recoveries for different plant matrices (93–120%) [82].

Neutral DTCs can be analysed separately by LC-MS with a source operating in atmospheric pressure chemical ionisation (APCI) in positive ion mode (APCI+). This ionisation resulted in more sensitivity than electrospray ionisation (ESI) in positive mode (ESI+) [83] (Table 1).

In a study of [84], it validated a quick but sensitive method to determine propineb (a representative from the PBDC group) and mancozeb (the representative of ethylenebis-dithiocarbamate EBDC group) simultaneously in real samples of fruits, vegetables and mushrooms. The analytes were decomposed in an alkaline medium and derivatised with dimethyl sulphate to PBDC-dimethyl and EBDC-dimethyl. After the extraction and cleaning with QuEChERS (quick, easy, cheap, effective, rugged and safe), the methyl derivatives of PBDC and EBDC were analysed with UHPLC-MS/MS and electrospray ionisation. The method allowed good recoveries in the samples examined (from PBDC to PBDC-dimethyl: 86.1–106.9%, while from EBDC to EBDC-dimethyl: 85.2–101.6%). The analysis of PBDC-dimethyl derivatives showed a LOQ of 0.5–1.5 µg/kg, while LOQ was 0.4–1.0 µg/kg for EBDC-dimethyl derivatives. In addition, the stability of the methyl derivatives of the two groups of fungicides was observed both in the extraction solvent (acetonitrile) and in the plant matrices [84].

Other researchers [85] characterised and analysed fungicides N,N–ethylene-dithiocarbamate (manzeb, maneb and zineb) in environmental water samples. The analytes were extracted in chloroform–hexane (3:1) and derivatised with methyl iodide. The derivatised products were determined in LC-MS with ESI ionisation, and the average recovery at the sub-ppb level was 79% (Table 1).

**Table 1:** Analytical methods used for the analysis of dithiocarbamates in different matrices.

*Researched Active Principle*	*Investigated Matrix*	*Principle of the Method*	*Equipment*	*LOD and LOQ*	*References*
*DTCs as sum of CS_2_*	Fruits and vegetables	DTCs are reduced to CS_2_	LC-MS/MS	LOD: 0.02–1.19 mg/kg LOQ: 0.03–2.69 mg/kg	[86]
*DTCs as sum of CS_2_*	Soy (leaves, pods, seeds, soil)	DTCs are reduced to CS_2_	GC-MS	LOQ: 0.32, 0.18, 0.19, 0.1 mg/kg	[87]
*Maneb*, *Zineb, Propineb Mancozeb*	Water and soil	DTCs are reduced to CS_2_ complexed with a copper acetate solution in the presence of diethanolamine	Spectrophotometer	n.a.	[88]
*Mancozeb*	Chamomile	The preparation involves the use of QuEChERS	LC-MS/MS	LOQ: 0.05 mg/kg	[89]
*Propineb, mancozeb, Thiuram*	Beer, malt and fruit juice	DTCs are methylated and subsequently analysed	LC-MS/MS	LOQ: <0.007 mg/kg	[90]
*DTCs as sum of CS_2_*	Foods	The evolved carbon disulfide is collected and reacted to form the yellow cupric salt of N,N-bis(2-hydroxyethyl) dithiocarbamic acid which can be measured colorimetrically	Spectrophotometer	n.a.	[91]
*DTCs as sum of CS_2_*	Lettuce	The purpose of this study was to compare the performance of GC-ECD, GC-PFPD and GC-MS and UV-VIS spectrophotometric methods	GC-ECD GC-PFPD GC-MS Spectrophotometer	LOD: 0.02–0.28 mg/kg LOQ: 0.05–0.40 mg/kg	[61]
*DTCs as sum of CS_2_*	Soybean	DTC are determined as CS_2_ using acidic hydrolysis and isooctane partitioning, followed by GC-PFPD and GC-ITD-MS analyses	GC-PFPD GC-ITD-MS	LOD: 0.02 mg/kg LOQ: 0.05 mg/kg	[75]
*DMDC-methyl* *EBDC-dimethyl*	Tap water	The samples were prepared by a modified version of the pre-treatment method for polycarbamate analysis by HPLC	TPI on-column GC/MS	LOQ: 0.3 g/L	[76]
*Milneb*	Foods	DTCs and milneb were extracted from foods with cysteineῌEDTA solution as sodium salts, and methylated with methyl iodide	GC-MS	LOQ: 0.01 mg/kg	[77]
*Ziram*	Spinach	First method: extraction of 1 g of lyophilised sample with a 1:1 (*v/v*) aqueous EDTA-methanol solution and later partition with hexane as clean up; Second method: involves supercritical carbon dioxide, with the addition of methanol as an organic modifier, to perform the extraction of 0.25 g of lyophilised sample	HPLC-UV	LOQ: 0.05 mg/kg	[80]
*N-methyl-DTC* *N,N-dimethyl-DTC Ethylenebis-DTC Propylenebis-DTC*	Fruits and vegetables	A new reversed-phase ion-pair chromatographic method was developed, consisting of surface extraction followed by direct injection into a liquid chro- matographic system equipped with UV and electrochemical detectors, connected in series	LC-UV LC-ED	LOD: 4–7 µg/L LOQ: 8–18 µg/L	[82]
*EBDC,* *PBDC*	Fruits, vegetables and mushrooms	EBDCs and PBDCs were decomposed in an alkaline medium and derivatised with dimethyl sulfate to EBDC-dimethyl and PBDC-dimethyl, respectively	UPLC-MS/MS	LOQ: 0.0004–0.0015 mg/kg	[84]
*Manzeb, Maneb, Zineb*	Environmental water	EBDCs were transformed into water-soluble sodium salts by adding an alkaline EDTA solution. Subsequently extraction and derivation are carried out	LC-MS	LOD: 0.043 µg/L	[85]
*Disulfiram*, *Dazomet, Thiram*, *Metabolites*	Fruits and vegetables	First method: MSPD + LC-APCI- MS Second method: SPE + LC-APCI-MS	LC-APCI-MS	LOQ: 0.25–2.5 mg/kg	[92]

### 6.4. SPE Extraction

The choice of the right DTC analysis technique, as for other pesticides, depends on the chemical–physical properties of the molecules and obviously on the economy, instrumental and labour resources available. The analysis of DTC fungicides has seen the transition from spectrophotometric to spectroscopic techniques to the success of chromatographic methods (HPLC-MS or GC-MS). In fact, the latter show optimal instrumental performance such as sensitivity and accuracy, achieving at the same time satisfactory LOQ.

However, the success of these techniques depends on the complexity of the matrix of the sample and the chemical–physical characteristics of the molecules belonging to the DTCs group. Therefore, in HPLC-MS or GC-MS analyses, cleaning and extracting analytes become essential to eliminate all interferences in food and environmental matrices. Since environmental and food samples can contain many components with different properties, the matrix′s complexity will affect the chromatographic techniques′ robustness [2].

The analysis of these fungicides requires a sample preparation step to remove the compounds that cannot be quantified chromatographically to avoid the matrix effect. For this reason, many authors, in their studies, refer to techniques of liquid or solid phase extraction (SPE). In this way, it is possible to increase the concentration of analytes of interest and significantly improve the sensitivity of the determination. Some authors have used liquid–liquid partitioning with dichloromethane or SPE on Extrelut columns, often obtaining poor recoveries [92].

In a study of [93], it was defined a simple method for analysing fungicidal polycarbamate in riverine and tap waters. The samples were placed in an alkaline solution of EDTA/cysteine and then subjected to SPE with the Oasis HLB cartridges (60 mg, 3 mL), eluting the final extract with distilled water. The LC-MS/MS analysis in positive atmospheric pressure ionisation followed the extraction. The LOD and LOQ for DMDC-methyl in water were, respectively, 0.061 and 0.21 µg/L. Otherwise, the analysis of EBDC-dimethyl showed LOD and LOQ of 0.032 and 0.11 µg/L, respectively.

Another study of [92] optimised two different extraction protocols to evaluate the best approach in terms of recovery in the analysis of DTCs and their metabolites (propilenthiourea or PTU, etilenthiourea or ETU) in commercial vegetable matrices. The first protocol assessed the SPE extraction, while the second one was based on solid-phase matrix dispersion methods (MSPD). The authors prepared preliminary tests using solutions of fungicides at different concentrations in water in order to understand the influence of different parameters, including salt addition, pH, homogenisation of samples before extraction and the solid phase (florisil, alumina, C18, C8, carbon and silica) on the SPE. Differently, they tested the better dispersion agent (C8, C18 and carbon) for optimal extraction based on the MSPD procedure. Both extraction procedures demonstrated several advantages, such as poorly used organic solvent, low cost, small amount of sample and good sensitivity. However, as far as the recoveries are concerned, the MSPD showed better recovery values than SPE. On the one hand, the MSPD showed recoveries in the range of 5–90% for DTCs and 64–89% for metabolites. On the other hand, SPE recoveries ranging from 3% to 90% were obtained for DTCs and, lower than the MSPD method, for the metabolites. The metabolites′ high polarity and water solubility probably do not allow good recoveries with SPE. However, the SPE could increase the sensitivity of the determination due to the possibility of extracting a large amount of sample (up to 10 g), leading to an improved LOQ with, at the same time, good recoveries. In addition, the pH and salt addition did not show an important effect on the SPE extraction. Vice versa, the homogenisation of the sample influenced the extraction and the best results, in terms of recoveries, were obtained by homogenising the samples with water, probably due to the loss of the compounds during evaporation using solvents [92].

### 6.5. Alternative Analytical Approaches

An alternative analytical approach to DTC analysis concerns the electrochemical approaches. Several authors have deepened the application of electrochemical sensors as simpler and cheaper tools than spectroscopic and chromatographic techniques for analysing DTCs.

Using electrochemical instruments to determine DTCs has been a known technique for decades [94]. The DTCs lend themselves well to this analysis due to the presence of various electroactive sites on their surface. Even in water, their determination is favoured by the presence of thiol groups that dissociate from the metal, forming the carbamate anions. For example, in aqueous conditions, thiol groups of propineb lose interaction with zinc, forming their carbamate anion [95]. In the electrolytic analysis of DTCs, other factors such as the pH of the solvent electrolyte, the type of electrode and the waveform used for the analysis are involved in the control of the fraction of the molecule detected [35].

In this analytical context, the low solubility of these molecules in water improves the method′s sensitivity because a preconcentration of the analytes is to be determined before the quantitative phase takes place; this is because the molecules adsorb to the surface of the electrodes. Many methods studied used non-modified metal or carbon electrodes applying important voltages to determine DTCs effectively. The voltages could reduce the specificity of the signal due to the presence of other compounds that react electrolytically, influencing the signal and, thus, the selectivity of the method used. Despite the great attention shown towards these simple and economical methods, they cannot be used for routine analysis. In fact, electroanalytical methods do not have sufficient specificity and are conditioned by various interferences [35].

In order to optimise the separation process for complex mixtures to be analysed, two-dimensional chromatography is used. It leads to the enrichment and purification of analytes, increasing separation and method sensitivity. In a study of [96], the researchers conducted a monitoring study of prenatal exposure to mancozeb. The mothers′ urine was analysed to determine the metabolite ETU. Complex analysis was performed by two-dimensional liquid chromatography with a triple-quadrupole linear ion trap (QTRAP 5500; AB Sciex, Milan, Italy).

To improve analytical selectivity, emphasis has been placed on techniques involving the use of biosensors. These techniques associate a sensitive analysis method with a precise biorecognition element. They overcome the drawbacks of electrochemical sensors because they exploit electron transfer mediators or provide a direct passage of charges from enzymes to the electrode. Therefore, large voltages are not necessary.

Biosensors have been used more for other pesticides than DTCs due to the low commercial availability of molecular-compatible biological molecules (aptamers, antibodies and polymers). DTCs inhibit the enzymes laccase, tyrosinase and aldehyde dehydrogenase, and this ability has, therefore, been exploited to develop biosensors that use these proteins [97,98,99,100,101,102]. The application consists in exposing the sample (containing the active principle of the fungicide) to the sensor and measuring the signal before and after exposure. In the presence of DTCs that inhibit the activity of enzymes, the electrochemical signal is reduced proportionally to the amount of fungicides present [35].

The electrochemical detection method may be poorly selective because enzymes are inhibited by a group of DTCs but not by individual compounds belonging to this class of fungicides. Furthermore, other molecules can inhibit these proteins, e.g., heavy metals. Therefore, the detection by enzymatic inhibition based on biosensors could be used as a quick method to make a first screening. Several researchers are deepening their knowledge about them to solve the selectivity problem. Some authors investigated the potential use of multiplex sensors, others the use of enzymes extracted from more stable extremophiles and with greater specificity for the substrate. It is also interesting to use genetic engineering to create new enzymes that can be efficient for these applications. In addition to controlling enzymatic abilities, research today investigates new nanomaterials that can produce a better electrochemical signal. The goal is to create rapid, effective and sensitive devices to build biosensors suitable for both DTC analysis and sampling [35].

## 7. Analysis of Dithiocarbamate Metabolites

Ethylene thiourea (ETU) and propylene thiourea (PTU) are the main degradation products of dithiocarbamates. Their determination in water is very difficult due to their polar nature. It is known that different DTCs have different toxicological potentials. The parental compounds decompose, giving rise to more toxic metabolites.

The massive use of DTCs has led to the development of several analytical protocols to quantify their main metabolites ETU and PTU. Various extraction approaches were applied in the sample preparation, such as liquid–liquid extraction with dichloromethane and SPE on Extrelut and reverse-phase columns or MSDP. Different analytical methods have been tested, such as gas chromatography with electron capture and nitrogen-phosphorus detection or high-performance liquid chromatography with multiple detectors [103].

In a study of [104], the degradation of propineb was studied, following its metabolism with field tests in soil and banana. Propineb releases propylenediamine (PDA) and PTU. The authors developed a simple but sensitive method for analysing propineb in food and environmental samples, including quantifying the fungicide by GC-FPD. For the metabolite analysis, sample preparation with QuEChERS was followed by analysis in LC-MS/MS.

Moreover, the researchers conducted field dissipation tests showing a half-life of approximately 13 days in the soil for propineb and approximately four days in the fruit. As for metabolites, PTU was detected in the banana as the main metabolite with a half-life between 32 and 69 days [104].

N-methyldithiocarbamate sodium was detected with its metabolite methyl isothiocyanate (MITC) in water. The analytes were analysed by HPLC coupled to UV detection at 242 and 284 nm. The separation of the analytes was performed on a column with strong anionic exchange with methanol/buffered water containing ammonium bromide hexadecylmeter (CTAB). Recoveries were 108% for the progenitor with a LOD equal to 2 mg/kg and 114% for the metabolite with a LOD of 2 µg/kg [105].

A quantitative analysis of the metabolites ETU and PTU was also carried out using UHPLC coupled to an amperometric detector by [106]. The latter works with a carbon electrode silkscreened with cobalt phthalocyanin (CoPc-SPCE). The separation took place on a C18 reverse-phase column in isocratic mode. The method was demonstrated to be simple and inexpensive for analysing these metabolites in fruit samples. Indeed, a LOD of 0.009 µg/kg for PTU and 0.006 µg/kg for ETU was reached [106].

The EU Reference Laboratories (EURLs) for Residues of Pesticide have validated a “Quick Method for the Analysis of Highly Polar Pesticides in Food” of Plant Origin (QuPPE-OP method) [107]. 

The LC method M4.2 covers the analysis of ETU and PTU in food of plant origins such as fruits, vegetables, cereals, dry pulses, oily seeds and honey. The DTCs metabolites and other pesticides are extracted with acidified methanol (1% formic acid) from a homogenized sample previously adjusted for the water content. The extract is then centrifuged, filtered and directly analysed by LC or IC-MS/MS [107]. Some authors tried the method for the ETU and PTU determination, obtaining good linearity in the 10–400 ng/g range with LOQ and LOD equal to 10 and 3.3 ng/g, respectively [108].

In addition to the studies for the analysis of PTU and ETU, there were analytical evaluations concerning two other metabolites. The methyl isothiocyanate (MITC) and N,N -dimethylthiourea (DMTU) are more toxic than dazomet and metam from which they derive. A study of [109] proposed an alkaline extraction and methylation with dimethyl sulphate for the analysis of PTU and propineb [109]. The procedure can also be applied to DMTU and other DTCs showing the same structure. Otherwise, for the analysis of MITC metabolite, further studies to confirm the method′s applicability are necessary.

The determination of MITC may be difficult due to the volatile nature of isothiocyanates, which makes gas chromatography the most suitable technique for their detection. However, studies show that approximately 80% of compounds degrade during GC analysis into new molecules, causing wrong results [5].

Some authors tried the detection of MICT by HPLC-UV-VIS using a cyclocondensation reaction with 1,2-benzenedithiol and a coloured derivative [110]. However, the reaction estimates the total content of isothiocyanates, not allowing the differentiation of MITC from other isothiocyanates present in the sample.

In another study, the researchers developed in 2017 an analytical protocol for the quantitative and qualitative determination of isothiocyanates, including MITC. The method involves isolating and cleaning analytes with SPE, followed by derivatisation with N-acetyl-l-cysteine (NAC) and separation with HPLC coupled to diode detector and mass spectrometer (HPLC-DAD-MS). The authors obtained good results in terms of linearity within the ranges of 0.005–0.1 μmol/mL and 0.1–1.0 μmol/mL for the determination of isothiocyanates in different crops such as white cabbage, broccoli, papaya, etc. Good recoveries were also obtained, ranging from 83.3 to 103.7%, with relative standard deviations lower than 5.4% [111].

## 8. Monitoring of Dithiocarbamates

The presence of DTCs in biological, environmental and food samples requires considerable attention to the toxicological aspects that these fungicides can impact both on humans and the environment. Regarding human exposure to DTCs and, in general, to all pesticides, occupational exposure of workers and public exposure of the population through the diet should be considered. In fact, the wide use of plant protection products leads to significant residues in food. For this reason, government authorities have established over time maximum residue limits (MRLs) for DTCs, banning some of them to ensure food safety. Continuous monitoring of DTC residues in food is a valuable tool for efficient risk assessment. The DTCs were the most relevant class of pesticides monitored in the food samples examined during two important campaigns in Brazil [112]. They were also the most observed pesticides in other global monitoring campaigns carried out in Canada and European countries [113,114].

After application, a significant portion of the pesticides applied is found to remain associated with the soil and food over long periods of time. The EU legislation (Regulation (EC) No 396/2005) foresees monitoring pesticides in foods on the European market. In order to achieve this purpose, the European Food Safety Authority (EFSA) provides an annual report which analyses pesticides in food. The last reports [115,116,117,118] show DTCs as the pesticides with the highest frequency of detections in organic food after copper compounds. Some DTC residues were quantified in levels exceeding their corresponding acute health-based guidance values (2018–2019). In EFSA′s latest report [115], DTCs were identified as the pesticides for which the maximum residue level (MRL) exceedance rate was above 1% (1.2%). The last quantification rate of DTCs resulted equal to 1.9%, mainly in roman rocket/rucola, table grapes and grapefruits.

In this context, monitoring pesticide levels in environmental and biological samples of humans and animals became essential to gather information on the overall effective exposure to these molecules (Table 2).

Some researchers carried out in 2010 a biomonitoring study to assess the concentration of ETU in the urine of the UK population. A total of 131 samples were examined to assess background levels of ETU in the English population. The ETU was found in 50% of the participants (males and females in equal numbers), with concentrations equal to those observed in other European countries [119] (Table 2).

ETU is considered an antithyroid molecule due to its toxicity to this gland [120]. Biomonitoring studies have been performed to assess the incidence of abnormal thyroid disease related to occupational exposure to the EBCD metabolite. In particular, in one study, it emerged that ETU in the blood is more valid as a biomarker for evaluating exposure to EBDCs than the same analyte found in the urine. Therefore, it is assessable to consider ETU as a valid biomarker in biological monitoring studies investigating thyroid disorders [121] (Table 2).

The ETU levels were also correlated with respiratory disorders in children′s first year of life. A biological monitoring campaign (‘Infantes y Salud Ambiental’, ISA) has been carried out in Costa Rica to assess the prenatal influence of some pesticides, including ETU, on respiratory diseases in children. The obtained data showed a correlation between ETU levels in the urine of pregnant women and the development of respiratory disorders in children, such as lower respiratory tract infections. However, further research should validate these results (Table 2) [96].

The monitoring tool is also used for assessing water resources using organisms as indicators of the qualitative status of the aquatic environment. In general, the aquatic environment represents the final collector of human activity. The evaluation of a water body′s chemical, physical and biological properties is an approach to assessing its quality. Some authors conducted in 2020 a biomonitoring study evaluating thiram′s short- and long-term toxicity on a freshwater bioindicator, *Daphnia magna*. This crustacean is often used as an organism sensitive to a range of environmental stresses to evaluate changes in the quality of a freshwater body. Chronic and acute toxicity tests have been carried out on this bioindicator. On the one hand, *Daphnia magna* was exposed to low concentrations of thiram (0.004–0.008–0.016–0.032 µg/kg) for 21 days in a chronic toxicity assay. On the other hand, the acute toxicity exposed the organisms to higher thiram concentrations (0.05–0.10–0.25–0.50 µg/kg) for 24.48 h. The results showed organism effects on reproduction, growth and neurotoxicity [122] (Table 2).

However, at the same time, they can be toxic to other living organisms exposed to these molecules.

In the literature, monitoring studies demonstrate the toxicity of specific DTCs in animals such as rodents in particular. Other authors, found that mancozeb causes thyroid and testicular disorders in these animals, but no liver toxicity was observed [123] (Table 2).

Very important are also the studies of biomonitoring about propineb, which demonstrated harmful effects on the environment and human health, suggesting also a synergistic reaction when combined with other pesticides [124]. Indeed, the study conducted on zebrafish (*Danio rerio*) observed the toxic effect of propineb on thyroid function and its exertion of immunotoxicity, cytotoxicity and neurotoxicity in humans. Zebrafish has more than 70% of genes associated with human diseases, and so it is a useful experimental model for investigating human diseases, development processes and toxicological effects [124]. In general, it has favourable characteristics for setting up chemical and genetic studies in vertebrates [125].

Acute toxicity tests have been performed according to the Organisation for Economic Cooperation and Development (OECD) guidelines for chemical testing [126]. Zebrafish embryos were exposed to different concentrations of propineb ranging from 17 to 0 mg/kg for a time of exposure of 72 h.

The toxic effects of the fungicide were detected by measuring the physiological and morphological abnormalities of organisms through a series of techniques such as microscopic observation with image processing with particular software (Image J, Bethesda, MD, USA) and colouring TUNEL (deoxyucleotidyl terminal transferase dutp nick end labelling) to detect apoptosis. The analysis of gene expression and the creation of transgenic organisms to control the vascularisation and growth of blood vessels during embryo formation was also carried out. The collected data clearly demonstrated the toxicity of the fungicide [127] (Table 2).

Finally, ETU was also found in plants treated with the fungicide maneb. Treatments of different plant cultures with isotopic labelled ETU [4,5-^14^C] were used to study its metabolism. The molecule was transferred throughout the plant by the xylem, and, 20 days after treatment, only approximately 1–2% of the initial dose was found. However, other similar metabolites, such as ethylene urea (EU), resulted from its degradation [128,129] (Table 2).

**Table 2:** Dithiocarbamate monitoring studies assessed in human, food and animal samples.

**Human Monitoring**					
**Sample Type**	**Analytes**	**Analytical Method**	**Time of Monitoring**	**Monitoring Results**	**References**
4727 people (ages 1–79) Survey on health effects of human fruit and vegetable consumption containing residues of DTCs/ETU and other pesticides	DTCs/ETU	Risk/benefit assessment through the use of real databases and mathematical models.	n.a.	❖ DTCs identified as priority pesticide active ingredients; ❖ DTCs contribute to cancer and non-cancer risk; ❖ A cancer risk greater than one extra lifetime case of cancer over 100,000 persons which is the maximum tolerable risk obtained for DTCs.	[114]
Urine	ETU	APCI-LC-MS	Approximately 2 months	❖ 54% of samples: ETU levels < LOD; ❖ Maximum value 14.3 ppb of creatinine; ❖ 50% of the English population have ETU levels similar to other countries in Europe.	[119]
Blood, urine and environmental samples	ETU	HPLC-UV for ETU/medical examinations for exposed, no exposed and control workers	Approximately 1 year	❖ ETU levels in air and soil < to authorisation levels according to U.S. EPA; ❖ Significantly different ETU levels in blood in exposed, unexposed workers and in control; ❖ Significant correlation nodule size and blood ETU (*p*-value 0.001); ❖ Not-significant correlation of urine in exposed workers, not exposed and in control (*p*-value 0.10).	[121]
Maternal urine	Mancozeb/ETU	LC-MS/MS	15 months	❖ 39% of children: an episode of dyspnoea during the first year of life; ❖ 10% of children: an episode of lower respiratory tract infection during their first year of life.	[96]
**Food Monitoring**					
**Sample Type**	**Analytes**	**Analytical Method**	**Time of Monitoring**	**Monitoring Results**	**References**
Fruit and vegetable	DTCs and other pesticides	CS_2_ analysis with spectrophotometry/gas chromatography (GC/FPD)	10 years (from 2001 to 2010)	❖ DTCs were present in 41.6% of the examined foods.	[112]
Food (biological and animal food; baby and young food)	DTCs and other pesticides	Evaluation and statistical analysis of data derived from official controls of the Member States of the European Union, of Norway and Iceland	1 year (2018)	❖ In general, residues of DTCs and other pesticides in the food concerned are not a food safety hazard.	[118]
Fruit (tangerines, oranges, peaches, nectarines, khakis)	DTCs	CS_2_ analysis with GC-MS	20 months	❖ DTCs were the most present residues. They were found in 37 samples (approximately 32% of contaminated samples).	[113]
Young seedlings and leaves of corn, lettuce, pepper and tomato	[4,5-^14^C] ETU	Liquid scintillation counting	more than 20 days	❖ 1–2% of the initial dose was found 20 days after treatment; ❖ EU resulted from its degradation in pepper plants.	[128]; [129]
**Animals Monitoring**					
**Sample Type**	**Analytes**	**Analytical Method**	**Time of Monitoring**	**Monitoring Results**	**References**
*Daphnia magna*	Thiram	Acute and chronic toxicity tests	21 days	Increase in the immobilisation rate; ❖ Decrease in different growth and reproductive parameters; ❖ Neurotoxic effect.	[122]
Zebrafish (*Danio rerio*)	Propineb	Acute toxicity tests [126]	72 h	❖ Morphological and physiological defects in embryos; ❖ Apoptosis early in development Deformation of the notochord in the larvae; ❖ Angiogenic insufficiency in transgenic larvae.	[127]
Male rats	Mancozeb	Histological studies and Hormone assay and liver function test	4 weeks	❖ Structural and functional changes in the male reproductive organ; ❖ Reduction of estradiol, progesterone and serum testosterone; ❖ Thyroid dysfunction; ❖ Liver toxicity has not been verified.	[123]
Wistar Rats	Mancozeb	Comet assay in total blood and the micronucleus test in bone marrow	18 days	❖ DNA damage; ❖ Genotoxic power.	[130]

n.a.: not available.

## 9. Conclusions

The presence and fate of dithiocarbamate residues and their degradation products in the environment, as well as their accumulation in edible products intended for human consumption and biological samples (e.g., blood, urine, etc.), are discussed in this study.

Several studies evidenced adverse health effects on humans arising from dermal contact through the skin, inhalation and ingestion of contaminated food; the prolonged exposition can lead to dermatitis, sensitisation and inflammation in the eyes and respiratory tract, skin allergy, as well as more severe effects such as functional changes in the nervous and hepatobiliary systems, hormonal and reproductive disorders, immunotoxicity and cytotoxicity were observed in previous studies.

The toxicity of dithiocarbamates is also attributed to their metabolic products, such as ethylene and propylene thiourea (ETU and PTU) metabolites. Indeed, ETU and PTU resulted in particular toxicological concerns due to their effects on reproduction and their tendency to form during food processing. Moreover, they are not covered by common monitoring programs.

Regarding the analytical approaches to be used, the integrity of the DTC during sampling, a proper pre-treatment and a sensitive, quantitative analysis ensuring the determination of single compounds must be guaranteed. Detailed knowledge of the potential chemical interactions of DTCs and the matrix in which they are suspended is essential for providing accurate analytical results.

New promising analytical approaches based on biosensors which exploit the enzymatic inhibition of DTCs demonstrated great potential as simple and cheap analytical tools.

Most of these compounds, used mainly as fungicides against a wide variety of fungi on many crops, are subjected to different EU PPP regulation. Zineb was never approved in the EU (2001), propineb and thiram were banned in 2018, maneb in 2017, while metiram is on the way of non-renewal in accordance with Regulation (EC) No 1107/2009 [131], and amending Commission Implementing Regulation (EU) No 540/2011 [132]. Otherwise, mancozeb is registered on the market from decades ago (since 1961) [37,133] but is not anymore approved in EU [37].

Recently, two significant regulatory acts, the Sustainable Use Regulation (SUR) and Nature Restoration Law (NRL), proposed by the European Commission, are under discussion in the European Parliament and promote practical and effective conservation of terrestrial and marine biodiversity in Europe in the near future, with significant consequences on the environmental sustainability of agricultural production and fishing.

In particular, the SUR proposes a new Regulation on the Sustainable Use of Plant Protection Products, including EU-wide targets to reduce by 50% the use and risk of chemical pesticides by 2030, in line with the EU’s Farm to Fork and Biodiversity strategies. Among the other key measures, the SUR promotes:

❖ an environmentally friendly pest control based on pest prevention and prioritises alternative pest control methods, considering the chemical pesticides option as a last resort;
❖ a ban for all plant protection products in sensitive areas such as urban green areas (e.g., public parks, gardens, playgrounds, recreation or sports grounds, public paths), protected areas that fall within the network of Natura 2000 sites and any ecologically sensitive area for pollinators;
❖ support farmers and professional pesticide workers to choose alternative and sustainable pest-control methods.

In a recent letter published in June 2023 [134], scientists supported the EU′s Green Deal, rejecting the argument against the SUR and NRL.

In summary, the researchers answered the first claim of opponents to the hypothetical yield production reduction with consequences for food security, stating that *“Protecting and restoring nature, and reducing the use of agrochemicals and pollutants, are essential for maintaining long-term production and enhancing food security”*.

As mentioned by [135,136,137], climate change and the loss of biodiversity and ecosystem services such as pollination and pest control are the greatest threats to food security. Fifty per cent of land cultivated depends on pollinators, and even more is subjected to anthropic pressures deriving from plant protection products, habitat destruction and climate change. A sustainable agrifood system based on functional diversity and a vegetation cover stabilising the microclimate, supporting pest control and pollination and reducing soil erosion is essential [138,139].

## Data Availability

Not applicable.
